# Referrals to chiropractors and osteopaths: a survey of general practitioners in rural and regional New South Wales, Australia

**DOI:** 10.1186/2045-709X-21-5

**Published:** 2013-01-31

**Authors:** Jon L Wardle, Davi W Sibbritt, Jon Adams

**Affiliations:** 1 Faculty of Health, University of Technology Sydney, 235-253 Jones St, Ultimo, NSW, 2007, Australia; 2 Network of Researchers in the Public Health of Complementary and Alternative Medicine (NORPHCAM), http://www.norphcam.org

**Keywords:** Chiropractic, Osteopathy, General practice, Rural healthcare, Health services, Referral, Interdisciplinary care, Primary care

## Abstract

**Background:**

Chiropractic and osteopathy form a significant part of the healthcare setting in rural and regional Australia, with national registration of practitioners, public subsidies for services and high utilisation by the Australian public. However, despite their significant role in rural and regional Australia, there has been little exploration of the interface between chiropractic and osteopathy and conventional primary health care practitioners in this area. The study aim was to examine the referral practices and factors that underlie referral to chiropractors and osteopaths by rural and regional Australian general practitioners (GPs), by drawing on a sample of GPs in rural and regional New South Wales.

**Methods:**

A 27-item questionnaire was sent to all 1486 GPs currently practising in rural and regional Divisions of General Practice in New South Wales, Australia.

**Results:**

A total of 585 GPs responded to the questionnaire, with 49 questionnaires returned as “no longer at this address” (response rate: 40.7%). The majority of GPs (64.1%) referred to a chiropractor or osteopath at least a few times per year while 21.7% stated that they would not refer to a chiropractor or osteopath under any circumstances. Patients asking the GP about CAM (OR=3.59; CI: 1.12, 11.55), GP’s use of CAM practitioners as a major source of information (OR=4.39; 95% CI: 2.04, 9.41), lack of other treatment options (OR=2.41; 95% CI: 1.18, 5.12), access to a wide variety of medical specialists (OR=12.5; 95% CI: 2.4, 50.0), GP’s belief in the efficacy of chiropractic and osteopathy services (OR=3.39; 95% CI: 2.19, 5.25) and experiencing positive results from patients using these services previously (OR=1.67; CI: 1.02, 2.75) were all independently predictive of increased referral to chiropractic and osteopathy services amongst the rural GPs.

**Conclusions:**

There is a significant interface between chiropractic and osteopathy and Australian rural and regional general practice in New South Wales. Although there is generally high support for chiropractic and osteopathy among Australian GPs, this was not absolute and the heterogeneity of responses suggests that there remain tensions between the professions. The significant interface between chiropractic and osteopathy may be due in part to the inclusion of these professions in the publicly subsidised national healthcare delivery scheme. The significant impact of chiropractic and osteopathy and general practice in rural and regional Australian healthcare delivery should serve as an impetus for increased research into chiropractic and osteopathy practice, policy and regulation in these areas.

## Background

The rising use of complementary and alternative medicine (CAM) - a diverse group of health care practices not considered part of conventional medicine - has emerged as a significant public health issue in Australia over recent decades, with visits to CAM practitioners accounting for up to half of all health consultations [1]. The professions of chiropractic and osteopathy form a significant part of the Australian CAM practitioner sector. Chiropractors are the second largest CAM profession in Australia, after naturopaths [2,3]. Although smaller than chiropractic, osteopaths were the fastest growing CAM profession in Australia in the ten years between 1996 and 2006 [3]. Between them, chiropractors and osteopaths represent 45% of the ‘primary-care capable’ CAM practitioner workforce in rural and regional New South Wales [2]. In addition to their significant presence in terms of practitioner numbers, both chiropractic and osteopathy professions are being increasingly integrated into healthcare delivery in Australia [4], with practitioners in both professions recently being included in the Australian national registration scheme for health practitioners [5], as well as consultations with practitioners in both professions being and eligible for public subsidies (Medicare) for their services [6].

General practice is one branch of medicine where CAM is making its presence felt, with data from Australian surveys indicating significant levels of referral from general practitioners (GPs) to CAM practitioners [7-9]. Whilst these studies indicate that GP referral to other GPs practising CAM therapies is more common than referral to those trained specifically in CAM modalities [9], there is also evidence of the development of a closer working relationship between CAM practitioners and GPs [7,8]. However, CAM integration is still an evolving and highly contested practice issue within primary health care [10,11] and the trend has been towards uneven integration with different therapies attracting different levels of support from GPs [7].

More specifically, chiropractic and osteopathy are major forms of CAM that are highly utilised by the Australian public, with surveys indicating utilisation rates estimated to be between 16% and 26% [12-15]. There also appear to be relatively high levels of support for chiropractic treatment amongst the Australian general practitioner (GP) community (compared to other CAM therapies), with a national survey indicating 72% of GPs rate chiropractic as moderately or highly effective while only 44% of respondents report the same of osteopathy [7]. However, issues such as the medico-legal implications of referral to CAM providers may constitute barriers to referrals arising from such high levels of support for chiropractic and osteopathy amongst Australian GPs [16]. Support for chiropractic and osteopathy amongst Australian GPs still appears, in part, to have translated into practice through significant referral patterns, with a national survey indicating 60% of Australian GPs regularly (at least a few times per year) refer to chiropractors, and 23% regularly refer to osteopaths [7]. These referrals form a significant part of the chiropractic and osteopathic patient load in Australia, with 20% of all chiropractic patients and 16% of osteopathic patients presenting via referral from a conventional medical practitioner [13].

Although there do appear to be high levels of support for chiropractic and osteopathy amongst Australian GPs, such support is not uniform and significant heterogeneity in GP attitudes towards these professions exists. Although a national survey of Australian GPs uncovered high levels of support amongst GPs for referral to chiropractic and osteopathic services, it also showed that a significant number of GPs would actively discourage patient use of chiropractic (16%) and osteopathy (21%) [7]. Medical practitioner support for chiropractic and osteopathic services may also be limited to their use in specific circumstances only; such distinctions were observed in a postal survey of Canadian GPs, which uncovered high levels of GP support for CAM in certain circumstances (such as chiropractic for musculoskeletal problems) but did not infer general support (such as chiropractic for general care, which was rated the lowest of all CAM for general health care treatment) [17].

Research is also uncovering geographical differences in CAM consumption both in Australian and internationally, with increased use by rural populations when compared to their urban counterparts [18]. Previous studies have shown high utilisation of chiropractic and osteopathy services in rural and regional Australia [12,14,19]. Additionally, in the Australian setting, the distribution of CAM practitioners in some regional and rural areas may be as great as conventional primary health care providers; with chiropractor and osteopath numbers being 40% of the numbers of GPs in rural and regional New South Wales [2]. However, rather than replacing conventional providers in underserved areas, CAM practitioner density appears to follow conventional provider density [2,20], and patients in rural areas may express dissatisfaction in the level of CAM services in their community as well as the level of conventional providers [21,22]. Research has also indicated that some CAM practitioners, such as chiropractors [23] appear to have a broader scope of practice and treat a wider variety of conditions in rural areas than they do in urban areas.

The high level of integration of chiropractic and osteopathy in the Australian healthcare sector, relative to other CAM professions, has significant implications for general practice and healthcare delivery in rural and regional Australia, especially when viewed in the context of higher use of these therapies in non-urban areas. However, despite the extensive presence of chiropractic and osteopathic practitioners, and the significant interactions that appear to occur between these practitioners and conventional medical providers, there has been little research to date exploring the level of integration at the grass-roots or the factors that underlie any integration of chiropractic and osteopathy in general practice in rural and regional Australia. This paper provides a first step in addressing this research gap by exploring the referral patterns of GPs in relation to chiropractic and osteopathy in rural and regional New South Wales, Australia.

## Methods

A 27-item questionnaire (Additional file 1) was mailed by post to all 1486 GPs registered as practising in rural and regional General Practice Divisions of NSW, with a reminder card sent after two months. The questionnaire was adapted for rural and regional use from previous Australian surveys of GP attitudes, use and practices of CAM [7,8]. The survey was piloted at the Department of General Practice, School of Medicine and Public Health, University of Newcastle, with modifications made based on feedback to ensure the instrument was clinically relevant. GPs were asked about their knowledge, attitudes, and practice and referral patterns to chiropractors and osteopaths and about CAM use in their areas more generally.

The final survey questionnaire contained 27 items which included multiple choice and multiple response close ended questions. The survey had five general areas: the GPs’ assumptions on chiropractic and osteopathy use by patients in their area; the GPs’ personal use and knowledge of chiropractic and osteopathy; the GPs’ professional relationship with chiropractic and osteopathy practice and practitioners; the GPs’ information seeking behaviours on chiropractic and osteopathy, and; the GPs’ specific opinions on chiropractic and osteopathy. GPs were also asked for demographic and practice information such as gender, age, number of years in practice, location of practice, number of patients seen per week and country of graduation. Ethical approval for the study was obtained from the School of Population Health Research Ethics Committee of the University of Queensland (JW130508) and the Human Research Ethics Committee of the University of Newcastle (H-2008-0344).

Rural and regional areas were defined by their classification in the Rural, Remote and Metropolitan Area (RRMA) classifications [24]. The RRMA classification categorises areas based on population and remoteness as large or small metropolitan (1–2), large, small and other rural centres (3–5); and remote or other remote (6–7). Rural and regional NSW was chosen as the study area due to research indicating high use of chiropractic and osteopathy in rural Australia [12,14,18,19], and further research indicating high prevalence of chiropractors and osteopaths, relative to other professions, in rural and regional NSW specifically [2]. To minimise the effects of local variation, every rural and regional GP in Australia’s largest state (New South Wales) was surveyed.

Questionnaire data was analysed using descriptive statistics via frequency distributions and cross-tabulations. Demographic and practice characteristics of GPs who referred to chiropractors and osteopaths often (at least monthly) and seldom or never were compared using chi-square tests. Logistic regression modelling, that included all practitioner and practice characteristics variables, was conducted using a backwards stepwise method of elimination using a likelihood ratio test, to parsimoniously predict referral to chiropractors and osteopaths. Statistical significance was set at the α=0.05 level. Data were analysed using the software program STATA 11.

## Results

A total of 585 questionnaires were returned completed, with 49 questionnaires returned uncompleted as ‘no longer at this address’; giving a response rate of 40.7%. Respondents had an average age between 45 and 54 years and were 53.5% male. Over three-quarters of respondents (77.8%, n=456) had completed their medical training at an Australian university. Aside from a slight over-representation of women, the respondent profile was broadly representative of the GP community in the study area in relation to average age and training location [25].

Referral rates of rural GPs to chiropractors and osteopaths are shown in the Table 1. One quarter (23.1%, n=135) of GPs referred to a chiropractor or osteopath at least once per month, with a further 41.0% (n=240) referring a few times per year. Most GPs were either actively referring to chiropractors and osteopaths, or would consider referring in the right circumstances, however approximately one-fifth (21.7%, n=127) of GPs stated that they would not refer to a chiropractor or osteopath under any circumstances. Most GPs were aware of local chiropractors and osteopaths in their area, with only 2.2% of respondents unable to identify practitioners to refer to.

**Table 1:** Referral rates of rural GPs to chiropractors and osteopaths in the past 12 months (n=585)

At least weekly	45 (7.7%)
At least monthly	90 (15.4%)
A few times per year	240 (41.0%)
I have not referred but would consider	70 (12.0%)
I would never refer	127 (21.7%)
I do not know of any practitioners	13 (2.2%)
No response	0 (0%)

Some GPs also perform chiropractic or osteopathic techniques themselves, with 10.4% (n=61) identifying that they had performed manipulative therapies on a patient during the past 12 months (data not shown). One-fifth (21.2%; n=124) of GPs had a personal professional relationship with a specific individual chiropractor or osteopath. More GPs had professional relationships with individual chiropractors (18.4%, n=108) than with individual osteopaths (5.6%, n=33).

Table 2 shows a comparison between GPs who referred to a chiropractor often (at least weekly or at least monthly) and seldom (less than a few times per year or never) by demographic characteristics. GPs were significantly more likely to refer to a chiropractor or osteopath if they had a high (over 151 patients per week) patient load (p<0.001). There was no significant association between referral to a chiropractor or osteopath and sex, age, level of rurality, nation of graduation from medical school and whether they had initially come from a rural area.

**Table 2:** Demographic and practice characteristics associated with referral to chiropractic or osteopathy by rural and regional GPs in New South Wales, Australia (n=585*)

		**Referral to chiropractic or osteopathy**		
**Demographic characteristics**		**Weekly or monthly**	**Seldom or never**	**p-value**
		%	%	
Sex	Male	50.9	58.1	0.096
	Female	49.1	41.9	
Age	25-34	8.3	10.0	0.539
	35-44	21.3	21.9	
	45-54	37.6	38.1	
	55-64	26.7	21.4	
	>65	6.1	8.6	
RRMA	3	27.5	30.0	0.067
	4	45.6	34.8	
	5	23.5	28.1	
	6	3.5	1.4	
	7	0.0	5.7	
Australian graduate?	Yes	77.6	78.6	0.786
	No	22.4	21.4	
Initially from a rural area?	Yes	36.3	25.2	0.066
	No	63.7	74.8	
Patient load (per week)	<50	17.3	13.8	0.001
	51-100	35.5	37.1	
	101-150	24.8	38.6	
	151-200	16.5	5.7	
	>200	5.9	4.8	

*except for RRMA, where n=579.

Table 3 shows a comparison between GPs who referred to a chiropractor often (at least weekly or at least monthly) and seldom (less than a few times per year or never) by other factors. Referral to a chiropractor or osteopath was significantly associated with level of knowledge about chiropractic or osteopathy (p<0.001), the number of patients asking about CAM (p<0.001), personal CAM use by the GP (p<0.001), patient request for referral (p<0.001), not having other options available (p=0.005), having had positive results with chiropractors or osteopaths previously (p<0.001), using CAM practitioners as a major source for CAM information (p<0.001), using patients as a major source for CAM information (p=0.001), belief in the efficacy of chiropractic or osteopathy (p<0.001), having prescribed CAM previously to patients (p<0.001) and being comfortable with referral to a chiropractor or osteopath (p<0.001).

**Table 3:** Other factors associated with referral to chiropractic or osteopathy by rural and regional GPs in New South Wales, Australia (n=585)

		**Referral to chiropractic or osteopathy**		
**Factors**		**Weekly or monthly**	**Seldom or never**	**p-value**
		%	%	
GPs level of knowledge of chiropractic and osteopathy	Excellent	3.5	7.1	<0.001
	Very Good	15.5	11.9	
	Satisfactory	54.3	37.6	
	Poor	26.1	34.3	
	Very Poor	0.8	9.1	
Percentage of patients who have asked about CAM	<10%	28.5	47.6	<0.001
	11-25%	4.1	44.8	
	26-50%	9.9	5.2	
	>50%	20.5	2.4	
GP’s personal use of CAM	Regularly	17.6	4.3	<0.001
	Often	15.7	21.4	
	Once/Rarely	35.2	22.9	
	Never, but would consider	15.2	9.1	
	Never, and would not consider	15.2	41.0	
Access to medical specialists is a problem	Yes	2.4	3.3	0.509
	No	97.6	96.7	
Patient request for referral	Yes	51.2	30.0	<0.001
	No	48.8	70.0	
Lack of other options	Yes	13.9	6.7	0.005
	No	86.1	93.3	
Positive results previously	Yes	58.7	30.5	<0.001
	No	41.3	69.5	
CAM practitioners are a major source of information on CAM	Yes	26.9	6.2	<0.001
	No	73.1	93.8	
Patients are a major source of information on CAM	Yes	52.3	38.1	0.001
	No	47.7	61.9	
Belief in efficacy	Yes	69.3	31.4	<0.001
	No	30.7	68.6	
GP interested in increasing CAM knowledge?	Yes	57.1	51.9	0.173
	No	42.9	48.1	
GP has prescribed CAM to patients previously	Yes	79.5	58.6	<0.001
	No	20.5	41.4	
CGP’s comfort level with chiropractic and osteopathy	Comfortable in general	30.9	4.8	<0.001
	Only in specific circumstances	52.4	22.9	
	Only if I knew them in person	14.0	14.3	
	I would not refer	1.9	58.1	

### Predictive factors

The result of multiple logistic regression modelling to determine independent predictive factors for referring to chiropractors and osteopaths is shown in Table 4. GPs who believed in the efficacy of chiropractic or osteopathy were 3.39 (95% CI: 2.19, 5.25) times more likely to refer to a chiropractic or osteopath at least once per month than those who did not. GPs who had seen positive results from chiropractic or osteopathy previously were 1.67 (95% CI: 1.02, 2.75) times more likely to refer to a chiropractor or osteopath at least once per month than those who had not and were 2.41 (95% CI: 1.18, 5.12) times more likely to often refer if they perceived there were no other options available. GPs who perceived access to medical specialists not being a driver for CAM use were 12.5 (95% CI: 2.4, 50.0) times more likely to refer to a chiropractor or osteopath more than once per month than those who did. No demographic factors were predictive for referral to chiropractors or osteopaths.

**Table 4:** Predictive factors for referral by GPs to chiropractic and osteopathy at least once per month by rural and regional GPs in New South Wales, Australia (n=573)

**Factor**		**Odds ratio**	**95% CI**
Percentage of patients who have asked about CAM	<10%	1.00	─
	11-25%	2.48	1.41, 4.37
	26-50%	7.64	3.54, 16.47
	>50%	3.59	1.12, 11.55
Access to medical specialists is a problem	No	1.00	─
	Yes	0.08	0.02, 0.41
Lack of other options	No	1.00	─
	Yes	2.41	1.18, 5.12
Positive results previously	No	1.00	─
	Yes	1.67	1.02, 2.75
Belief in Efficacy	No	1.00	─
	Yes	3.39	2.19, 5.25
CAM practitioners a major source of information on CAM	No	1.00	─
	Yes	4.39	2.04, 9.41

## Discussion

This is the first focused examination of conventional medical practitioner referral to chiropractors and osteopaths in rural and regional Australia. Our study findings show that a significant level of interaction exists between GPs and chiropractors and osteopaths in this area. The high prevalence of personal professional relationships and referral between GPs and chiropractors and osteopaths may be indicative of high presence of practitioners in the study area, as previous research has identified chiropractor and osteopath numbers around 40% of GP numbers in rural and regional New South Wales [2].

However, the high level of professional relationships and referral amongst GPs with chiropractors and osteopaths may also be related to formal referral arrangements that exist for chiropractic and osteopathic services in the Australian public health care system. Chiropractic and osteopathic patients are eligible to receive a subsidy of $51.95 from the Australian government when referred by a medical practitioner under the Medicare Extended Care Plan (MBS Item Numbers 10964, 10966, 81345 and 81350; figures correct as at August 2012) [26]. This interpretation is supported by the findings of this study, which show that the numbers of patients enquiring with their GP about CAM is a significant predictive factor for GP referral to chiropractors and osteopaths.

Chiropractors and osteopaths are currently the only CAM practitioners eligible for such subsidies in Australia. Formalised arrangements and subsidies for chiropractic and osteopathic services may therefore provide a cost-effective avenue (from the patient’s perspective) for GPs to explore CAM approaches to healthcare when such referrals are requested by patients, rather than being solely indicative of support for chiropractic and osteopathic treatments. Previous research on naturopaths in rural Australia, for example, indicates that although patients have a high level of support for CAM services, rural patients are often unable or unwilling to pay fully out-of-pocket costs associated with these services, and actively seek more cost-effective ways to access the advice of CAM practitioners [27]. With the introduction of other CAM professions into Australia’s national regulation scheme (Chinese medicine has already been included from July 2012, and naturopaths have been recommended for later inclusion [28]), there may be pressure to publicly subsidise these services as well, removing the current monopoly chiropractors and osteopaths regarding publicly-subsidised CAM services in Australia.

However, the issue of how government reimbursements or formalized arrangements affect referral patterns between conventional and specific CAM providers has not been explored in depth. Such exploration may be warranted, particularly as chiropractic more than any other profession appears to be driving high CAM practitioner use in rural areas in Australia [12,14]. Whether patient requests to GPs (and subsequent referrals by GPs) are specifically for chiropractic and osteopathy services, or whether the formalised arrangements mean that chiropractors and osteopaths simply provide a convenient avenue for GPs to refer to a CAM provider, will have a significant impact on which services GPs choose to refer to as more practitioner options become available. The finding from this study that GPs who rely on CAM providers as major sources of CAM information may not only indicate higher levels of interaction with CAM providers amongst referrers than non-referrers, but also that GPs are willing to communicate and refer to a broad variety of CAM practitioners beyond chiropractors and osteopaths. With medical referrals comprising nearly one-fifth the patient load for Australian chiropractors and osteopaths [13], increasing subsidised access to other CAM professions could have significant impacts on the professions of chiropractic and osteopathy, with reductions in referrals one possible scenario as GPs share their formal CAM referrals amongst a broader range of practitioners. Whilst this may benefit patients in terms of improved access to a broader range of therapeutic options, it could also pose professional challenges to the chiropractic and osteopathy communities.

Although our study results suggest that chiropractors and osteopaths may be largely accepted by the majority of the Australian rural and regional GP community, our findings also help identify remaining tensions between conventional medicine and the two CAM modalities, with one-fifth of GPs maintaining that they would never refer to a chiropractor or osteopath under any circumstances. This finding mirrors those of a previous national survey of Australian GPs which indicated both significant levels of support for, and opposition to, chiropractic and osteopathy referrals amongst the Australian GP community [7]. Such tensions have achieved recent attention in Australia, with calls from within the conventional medical sector for chiropractic and osteopathic practice to receive no further mainstream medical attention [29]. Findings from this study suggest that such ideologically opposed views to chiropractic and osteopathy do not seem representative of the majority of medical practitioners in our study area. However, significant tensions between the professions do highlight the need for further detailed research into factors that influence conventional practitioner opinion on CAM professions such as chiropractic and osteopathy.

However, other factors beyond ideological or professional opposition may also result in GPs being unwilling to refer to chiropractors and osteopaths, even if they exhibit positive attitudes towards the professions. Medico-legal concerns relating to GP referral to chiropractors have been disseminated to the practice community via a number of high profile court cases [16,30,31] and such concerns may be exacerbated by the focus of medical professional literature to the risks associated with CAM, rather than broader discussions of efficacy [32]. Indeed, previous Australian surveys of GPs have highlighted patient risk as a major determinant of GP opinion on CAM, often more than efficacy [7,8,33,34]. Further exploration of factors that make GPs less willing to refer to CAM practitioners (including osteopaths and chiropractors), as well as those factors that predict referral, would assist in providing further insights into the interface between CAM and general practice, the impact of CAM on primary health care, and the role that chiropractic and osteopathy can play in the broader health care system.

Rural and regional issues associated with patient CAM use and practice may also affect chiropractic referral, as some commentators have suggested that higher CAM use in rural and regional areas may be related to lower levels of conventional healthcare providers (e.g. specialists, allied health) in these areas [35]. Although a lack of other treatment options for patients was predictive of increased referral rates to chiropractors and osteopaths by rural and regional Australian GPs in our study, limited access to medical specialists was in contrast predictive of lower levels of referral. As such, increased referral to chiropractors and osteopaths may be related more to GPs referring to CAM after exhausting their own treatment options for patients, rather than serving as alternative referral recipients when specialist treatment is sought. This may be partly related to previous study findings that have suggested that rather than replacing conventional practitioners in areas of high need, CAM practitioner density often follows that of conventional practitioners, with areas of high service need experiencing shortages in both conventional and CAM practitioners [2,20]. Additionally, although previous large-scale surveys have highlighted a lack of access to or dissatisfaction with conventional medical services as associated with higher CAM use, such studies have also uncovered dissatisfaction in CAM service provider provision in rural areas [21,22]. Rather, other historical and cultural drivers (such as positive community connections, rural patient’s increased independence and stoicness, underlying community affinity for holistic principles and increased value of rural patients on experiential over empirical forms of evidence) may be push factors for CAM use by rural populations [18], which in turn may increase patient requests for CAM services and referrals for GPs in rural areas.

The high prevalence of professional relationships with individual practitioners may also be partly related to the rural and regional nature of the sample in this study, as smaller communities may facilitate increased interaction between CAM and conventional providers [18,19,36]. This may facilitate an increased level of referrals by rural and regional GPs as compared to their urban counterparts. Further investigation of referral patterns in the broader GP population, or comparative work with urban GPs, will assist in further ascertaining what role, if any, geographic factors such as the level of rurality have on the interface between CAM and general practice.

Though limited to one state (New South Wales), the large and varied study area was chosen to be broadly representative of Australian rural and regional general practice demographics [25]. Nevertheless, the demographics of the GPs in this study compared to national statistics (being as they are drawn from rural and regional areas and exhibiting a higher proportion of females) should be considered in generalising the study’s results to the broader Australian general practice population.

Other limitations of the study, in common amongst other questionnaire studies, include the use of self-reported data and possible recall bias inherent in retrospective collection of data over a 12 month period, as well as self-selection may also have resulted in some form of response bias. The response rate is typical for large-scale GP surveys on CAM conducted in Australia over the past decade, which have reported response rates of between 29.4-58.0% [7,33,37]. The response rate also compares well to general surveys of Australian GPs, which routinely have difficulty receiving response rates of over 30% [38].

## Conclusions

Our study reveals a high level of interaction (both via referrals as well as the development of professional relationships) between chiropractic and osteopathy and the GP community in rural and regional Australia. High use of chiropractic and osteopathy in the Australian community, combined with a high level of integration into conventional medical practice does highlight the important need for more research in this area, to ascertain the impact upon patient care delivery.

The significant presence, high utilisation and interface of chiropractors and osteopaths with rural primary health care should serve as an impetus for increased research into chiropractic and osteopathy practice, policy and regulation in these areas.

## Competing interests

The authors declare they have no competing interests.

## Authors’ contributions

JW was involved with conception and design of the study, collection of the data, analysing and interpreting the data and drafting and revising the manuscript; DS was involved with conception and design of the study, analysing and interpreting the data and drafting and revising the manuscript; JA was involved with conception and design of the study, analysing and interpreting the data and drafting and revising the manuscript. All authors read and approved the final manuscript.

## Supplementary Material

Additional file 1 Rural General Practice Complementary and Alternative Medicine Survey. Click here for file
