# Supplementary material for: Referrals to chiropractors and osteopaths: a survey of general practitioners in rural and regional New South Wales, Australia
Source: Chiropr Man Therap. 2013 Jan 31;21:5. doi: 10.1186/2045-709X-21-5 (PMC3599860; doi:10.1186/2045-709X-21-5)
Supplement: Additional file 1 — Rural General Practice Complementary and Alternative Medicine Survey. [file 2045-709X-21-5-S1.pdf]

THE UNIVERSITY OF QUEENSLAND
School of Population Health

THE UNIVERSITY OF NEWCASTLE AUSTRALIA
School of Medicine and Public Health
Department of General Practice

Rural General Practice Complementary and Alternative Medicine Survey

This project consists of two stages, this survey and follow up interviews. If you are interested in taking part in the second stage please fill in and mail the **separate contact form** provided or contact Jon Wardle at j.wardle@uq.edu.au

1. On average, what **percentage** of your patients do you **think** have used CAM in the past 12 months? ☐ %

2. On average, what **percentage** of your patients has **asked** you about CAM in the last month? ☐ %

3. Do you believe the demand from your patients for CAM is:
   Increasing ☐ Not Changing ☐ Decreasing ☐

4. Which of the following do you think **most** influences the use of CAM in your area? (Please tick all that are appropriate)
   Word of mouth ☐ Previous experiences ☐ Limited access to health professionals ☐ Good access to CAM therapists ☐
   Good communication between conventional and CAM practitioners ☐ CAM suited to local beliefs ☐ None of the above ☐

5. How would you rate your current level of knowledge of the following CAM?

	Excellent	Very Good	Satisfactory	Poor	Very Poor
Remedial Therapies/Massage	☐	☐	☐	☐	☐
Chiropractic/Osteopathy	☐	☐	☐	☐	☐
Naturopathy	☐	☐	☐	☐	☐
Herbal Medicine	☐	☐	☐	☐	☐
Nutritional Therapy	☐	☐	☐	☐	☐
Homoeopathy	☐	☐	☐	☐	☐
Acupuncture	☐	☐	☐	☐	☐
Traditional Chinese Medicine	☐	☐	☐	☐	☐
Spiritual Healing/Mind Body Medicine/Reiki	☐	☐	☐	☐	☐
Yoga/Meditation	☐	☐	☐	☐	☐

6. Are you interested in increasing your knowledge of CAM?
   Yes ☐ No ☐

7. Have you personally used CAM for your own health?

Regularly	Often	Once/Rarely	Never, but I would consider using	Never, and I don't intend to use
☐	☐	☐	☐	☐

8. Do you have a professional relationship with any specific CAM practitioners (for example referrals to or from, sharing of information etc)? If so please elaborate:

Yes ☐ No ☐

....
....
....
....

9. If you **would** refer, why do or would you refer to CAM practitioners? (tick all that apply)

Access to medical specialists is a problem	Patient requests for referral	Lack of other options	Have had positive results previously	Other Please elaborate
☐	☐	☐	☐	☐

....
....
....
....

10. How often would you refer to the following CAM Practitioners?

	At least weekly	At least monthly	A few times per year	I haven't referred but would consider	I would never refer	I don't know of any practitioners
Remedial Therapies/Massage	☐	☐	☐	☐	☐	☐
Chiropractic/Osteopathy	☐	☐	☐	☐	☐	☐
Naturopathy	☐	☐	☐	☐	☐	☐
Herbal Medicine	☐	☐	☐	☐	☐	☐
Nutritional Therapy	☐	☐	☐	☐	☐	☐
Homoeopathy	☐	☐	☐	☐	☐	☐
Acupuncture	☐	☐	☐	☐	☐	☐
Traditional Chinese Medicine	☐	☐	☐	☐	☐	☐
Spiritual Healing/Mind Body Medicine/Reiki	☐	☐	☐	☐	☐	☐
Yoga Meditation	☐	☐	☐	☐	☐	☐

11. On average, how often do you prescribe CAM to you patients?

At least weekly	At least monthly	A few times times per year	I haven't but I would consider	I would never prescribe CAM
☐	☐	☐	☐	☐

12. What CAM did you prescribe/practise **yourself**?

Remedial Therapies/Massage	☐
Manipulation (e.g. Chiro/Osteo)	☐
Naturopathy	☐
Herbal Medicine	☐
Nutritional Therapy	☐
Homoeopathy	☐
Acupuncture	☐
Traditional Chinese Medicine	☐
Spiritual Healing/Mind Body Medicine/Reiki	☐
Yoga/Meditation	☐

Other/specific treatments

....
....
....
....
....
....
....
....
....
....
....
....
....
....
....
....
....

13. If you do usually refer, where do you refer your patients for CAM products

	Often	Sometimes	Rarely	Never
Pharmacy	☐	☐	☐	☐
Health Food Store	☐	☐	☐	☐
CAM Practitioner	☐	☐	☐	☐
Stock in own clinic	☐	☐	☐	☐
Other GP clinic	☐	☐	☐	☐

14. Where do you receive most of your information on CAM? (Please tick all that are appropriate)

CAM Practitioners ☐ Other GPs ☐ Own Interest ☐ Patients ☐

Continuing Professional Education (CPE) ☐ Peer Reviewed Publications ☐ General Media ☐ None of these/ Other (please elaborate) ☐

....

15. Which sources of CAM information would you deem trustworthy?

	Consider Trustworthy	Neither Trustworthy nor Untrustworthy	Consider Untrustworthy
CAM Practitioners	☐	☐	☐
Other medical practitioners	☐	☐	☐
Self Interest/Research	☐	☐	☐
Patients	☐	☐	☐
Continuing Professional Education (CPE)	☐	☐	☐
Peer Reviewed Publications	☐	☐	☐
General Media	☐	☐	☐

16. In your opinion, which of the following CAM therapies do you consider to have:

	Ample Research/ No efficacy	Ample Research/ Good efficacy	Insufficient research/ No efficacy	Insufficient research/ Good efficacy
Remedial Therapies/Massage	☐	☐	☐	☐
Chiropractic/Osteopathy	☐	☐	☐	☐
Naturopathy	☐	☐	☐	☐
Herbal Medicine	☐	☐	☐	☐
Nutritional Therapy	☐	☐	☐	☐
Homoeopathy	☐	☐	☐	☐
Acupuncture	☐	☐	☐	☐
Traditional Chinese Medicine	☐	☐	☐	☐
Spiritual Healing/Mind Body Medicine/Reiki	☐	☐	☐	☐
Yoga/Meditation	☐	☐	☐	☐

17. How do you feel about potentially referring to the following practitioners:

	Comfortable In general	Only in specific circumstances	Only if I knew them in person	I would not refer
Remedial Therapies/Massage	☐	☐	☐	☐
Chiropractic/Osteopathy	☐	☐	☐	☐
Naturopathy	☐	☐	☐	☐
Herbal Medicine	☐	☐	☐	☐
Nutritional Therapy	☐	☐	☐	☐
Homoeopathy	☐	☐	☐	☐
Acupuncture	☐	☐	☐	☐
Traditional Chinese Medicine	☐	☐	☐	☐
Spiritual Healing/Mind Body Medicine/Reiki	☐	☐	☐	☐
Yoga/Meditation	☐	☐	☐	☐

18. How well do you feel the following therapies integrate with general practice (ie are compatible to be used together)?

	Very well	Appropriate for integration	No opinion	Not appropriate for integration
Remedial Therapies/Massage	☐	☐	☐	☐
Chiropractic/Osteopathy	☐	☐	☐	☐
Naturopathy	☐	☐	☐	☐
Herbal Medicine	☐	☐	☐	☐
Nutritional Therapy	☐	☐	☐	☐
Homoeopathy	☐	☐	☐	☐
Acupuncture	☐	☐	☐	☐
Traditional Chinese Medicine	☐	☐	☐	☐
Spiritual Healing/Mind Body Medicine/Reiki	☐	☐	☐	☐
Yoga/Meditation	☐	☐	☐	☐

19. Are there any further comments you'd like to add regarding CAM and rural general practice?

....
....
....
....
....
....
....
....
....
....
....
....
....
....
....
....

20. What is the RRMA classification of your primary clinic? ☐

21. Age: 25-34 ☐ 35-44 ☐ 45-54 ☐ 55-64 ☐ >65 ☐

22. Gender: Male ☐ Female ☐

23. Year of graduation: ☐ Years in general practice: ☐ Years in *rural* general practice ☐

24. Have you been given any specific CAM training?

Yes (**during** medical training) ☐ Yes (**before** medical training) ☐ Yes (**after** medical training) ☐ No training ☐

25. Were you originally from a rural area **before** entering rural practice?

Yes ☐ No ☐

26. Did you graduate in medicine from an Australian institution?

Yes ☐ No ☐ If no, what was your country of graduation? ☐

27. Average number of patients seen each week: <50 ☐ 51-100 ☐ 101-150 ☐ 151-200 ☐ >200 ☐

Thank you for your participation and completion of this survey.

Please place the completed survey in the reply paid envelope provided and send to:

Rural General Practice and Complementary Medicine Survey
School of Population Health
University of Queensland
Herston Q 4006
